# Digital Isolation and Dementia Risk in Older Adults: Longitudinal Cohort Study

**DOI:** 10.2196/65379

**Published:** 2025-02-19

**Authors:** Cheng Deng, Na Shen, Guangzhou Li, Ke Zhang, Shijun Yang

**Affiliations:** 1 Department of Cardiovascular Surgery, Union Hospital, Tongji Medical College Huazhong University of Science and Technology Wuhan China; 2 Department of Breast and Thyroid Surgery Union Hospital, Tongji Medical College Huazhong University of Science and Technology Wuhan China; 3 Department of Public Health University of Utah Salt Lake City, UT United States; 4 Department of Cardiology Union Hospital, Tongji Medical College Huazhong University of Science and Technology Wuhan China

**Keywords:** dementia, digital isolation, cognitive decline, older adults, elderly, geriatric, longitudinal cohort study, cognitive impairment, aging, social isolation, risk, digital engagement

## Abstract

**Background:**

Dementia poses a significant global health challenge, characterized by progressive cognitive decline and functional impairment. With the aging global population, dementia prevalence is projected to surge, reaching an estimated 153 million cases by 2050. While the impact of traditional social isolation on dementia risk has been extensively studied, the influence of digital isolation, a phenomenon unique to the digital age, remains underexplored.

**Objective:**

This study aimed to investigate the association between digital isolation and dementia risk among older adults, hypothesizing that higher levels of digital isolation significantly increase the risk of developing dementia.

**Methods:**

We conducted a longitudinal cohort study using data from the National Health and Aging Trends Study (NHATS), analyzing 8189 participants aged 65 years and older from the 3rd (2013) to the 12th wave (2022). Digital isolation was quantified using a composite digital isolation index, derived from participants’ usage of digital devices, electronic communication, internet access, and engagement in online activities. Participants were stratified into low isolation and moderate to high isolation groups. Dementia incidence was assessed using cognitive tests and proxy reports. Cox proportional hazards models were used to estimate the association between digital isolation and dementia risk, adjusting for potential confounders including sociodemographic factors, baseline health conditions, and lifestyle variables.

**Results:**

The moderate to high isolation group demonstrated a significantly elevated risk of dementia compared with the low isolation group. In the discovery cohort, the adjusted hazard ratio (HR) was 1.22 (95% CI 1.01-1.47, *P*=.04), while the validation cohort showed an HR of 1.62 (95% CI 1.27-2.08, *P*<.001). The pooled analysis across both cohorts revealed an adjusted HR of 1.36 (95% CI 1.16-1.59, *P*<.001). Kaplan-Meier curves corroborated a higher incidence of dementia in the moderate to high isolation group.

**Conclusions:**

Our findings indicate that digital isolation is a significant risk factor for dementia among older adults. This study underscores the importance of digital engagement in mitigating dementia risk and suggests that promoting digital literacy and access to digital resources should be integral components of public health strategies aimed at dementia prevention.

## Introduction

Dementia, characterized by progressive cognitive decline and functional impairment, represents a formidable global health challenge [1,2]. As the world’s population ages rapidly, the prevalence of dementia is projected to surge, with an estimated 153 million individuals affected by 2050 [3]. In the absence of curative treatments, the imperative for effective prevention strategies has never been more pressing. The etiology of dementia is multifactorial, encompassing both nonmodifiable factors such as age and genetic predisposition, and modifiable risk factors including cardiovascular health, lifestyle choices, and social engagement [4-6].

In the context of an increasingly digitalized society, interaction with technology has become integral to modern life. However, the digital revolution has not benefited all segments of the population equally. A significant proportion of older adults find themselves in a state of “digital isolation,” either due to limited access or inadequate digital literacy [7-9]. This concept extends beyond traditional social isolation by emphasizing the absence of digital engagement, including the use of the internet, smartphones, or social media, which can offer additional cognitive and social stimulation [10,11]. Recent studies indicate that older adults’ use of technology-based social platforms or electronic health resources may enhance cognitive outcomes, postpone cognitive decline, and alleviate loneliness. Consequently, individuals who are digitally isolated may miss these protective effects, which could accelerate cognitive decline and elevate their risk of dementia [11-13]. Limited literacy and education levels may further restrict older adults’ ability to engage with digital technologies, hindering their ability to benefit and potentially increasing the risk of cognitive decline [14,15].

While the association between traditional social isolation and dementia risk has been extensively studied, the impact of digital isolation, a phenomenon unique to our technologically driven era, has received comparatively little attention [16-18]. Preliminary investigations suggest a potential link between digital isolation and accelerated cognitive decline, as well as increased dementia risk. However, these studies are often constrained by limited sample sizes and cross-sectional designs, precluding the establishment of causal relationships. Furthermore, many existing studies fail to adequately control for potential confounding factors such as depression, anxiety, chronic comorbidities, and lifestyle variables, potentially biasing their results [19,20].

This study aims to clarify the relationship between digital isolation and dementia risk using a large-scale, longitudinal cohort design. Using a multistage Cox proportional hazards model on discovery and validation cohorts, we control for numerous potential confounders, including sociodemographic characteristics (eg, education level), baseline health status, and lifestyle factors. This study aims to provide strong evidence supporting the hypothesis that higher levels of digital isolation increase dementia risk. It also seeks to identify mechanisms underlying this emerging risk factor and to inform novel dementia prevention strategies, particularly those enhancing digital literacy and technology access. We aim to support a holistic public health approach that integrates traditional and digital aspects of social engagement in aging populations.

## Methods

### Study Population

This investigation used data from the National Health and Aging Trends Study (NHATS), a nationally representative longitudinal survey of Medicare beneficiaries aged 65 years and older in the United States. To ensure representativeness of the older adult population, appropriate survey weights provided by NHATS were applied to account for the complex sampling design. Our analysis used data spanning from the 3rd wave (2013), when digital product usage assessment was initiated, to the 12th wave (2022). The study cohort was stratified into discovery and validation samples.

In the third wave (2013), 5799 participants remained in the study. We excluded individuals lacking baseline digital isolation data or with preexisting dementia diagnoses. We rigorously controlled potential confounders influencing the digital isolation-dementia risk relationship, including age, education level, gender, race or ethnicity, baseline diseases, depression, anxiety, smoking status, and sleep difficulties. Participants were followed from the fourth wave (2014) through the twelfth wave (2022). During follow-up, some individuals were excluded due to attrition or death before dementia diagnosis. The final analytical sample comprised 4455 individuals.

**Figure 1:**
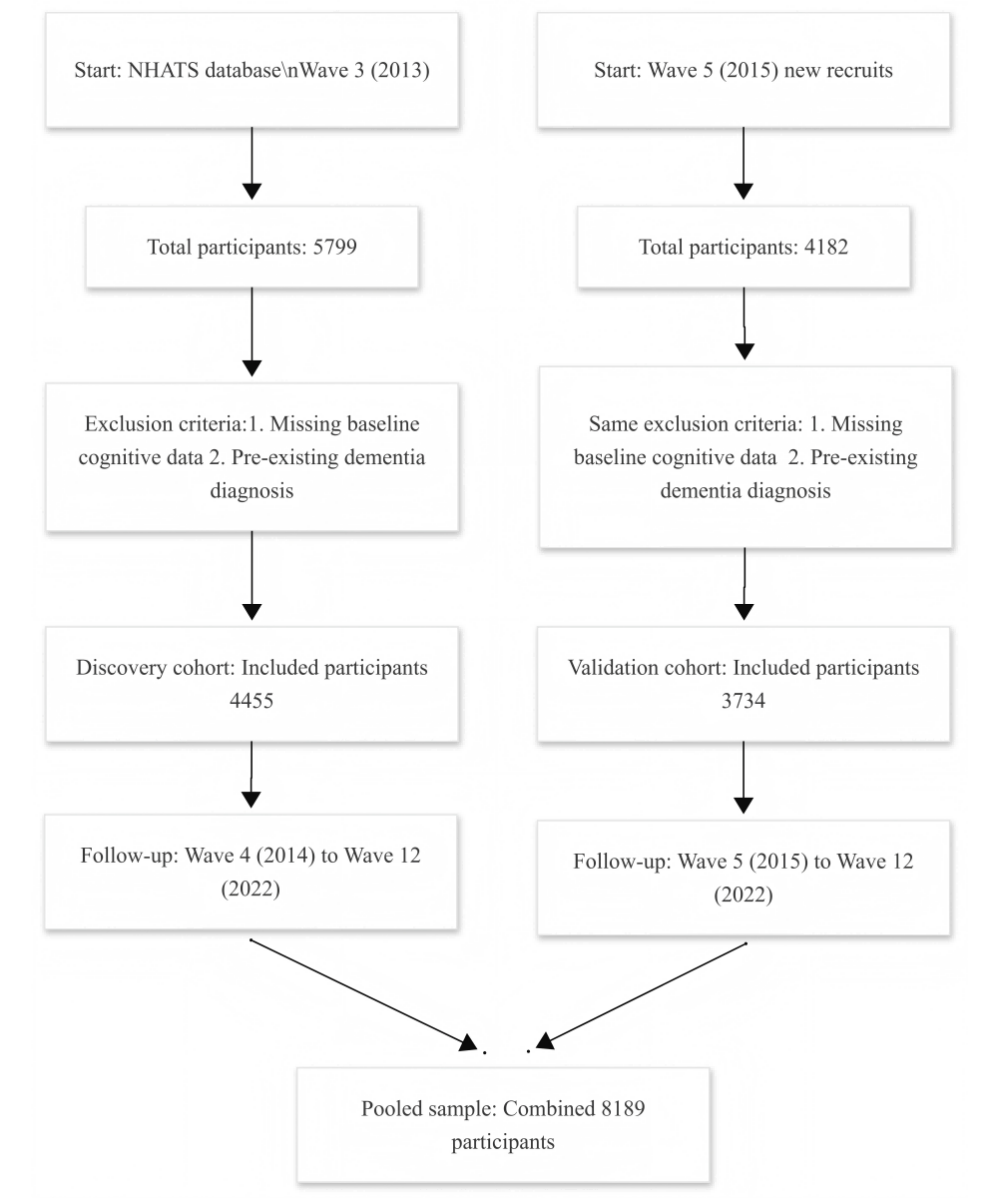
Flowchart depicting participant inclusion and exclusion criteria for the discovery (wave 3) and validation (wave 5) cohorts, with subsequent follow-up through wave 12 and formation of the pooled sample. NHATS: National Health and Aging Trends Study.

To validate our findings, we used an independent cohort of 4182 individuals newly recruited in the fifth wave (2015). After applying the same exclusion criteria as the discovery cohort and accounting for attrition and mortality during follow-up, 3734 individuals were included in the validation sample. This cohort was followed from the 5th wave (2015) through the 12th wave (2022). As shown in Figure 1, we provide a detailed overview of the inclusion and exclusion processes for the discovery and validation cohorts, including the study waves, exclusion criteria, and the final number of participants included in the analysis dataset.

### Digital Isolation

Digital isolation was assessed using a composite digital isolation index, which was constructed based on individuals’ usage of various digital devices and the internet. The design of this index was informed by relevant literature in the fields of social isolation and digital health. For instance, Cornwell and Waite [21] quantified social isolation by evaluating the extent of individuals’ social contact and participation and proposed a method for constructing a social isolation index using self-reported data. Similarly, digital isolation, as a modern form of isolation, is primarily reflected in the insufficient engagement with and use of digital technologies.

In this study, we operationalized digital isolation through a composite digital isolation index comprising 7 parameters (Textbox 1).

**Components of the Digital Isolation Index.**

**Parameters:**

- Mobile phone use.
- Computer usage.
- Tablet use.
- Frequency of electronic communication (email or text messaging).
- Internet access.
- Engagement in online activities.
- Participation in health-related digital platforms.

The selection and quantification of these indicators were informed by extant literature on digital technology adoption, such as Kraut et al [22], who investigated the impact of internet use on social involvement. Each parameter was dichotomized (0=nonuse, 1=use), and the sum of these binary scores constituted the aggregate digital isolation index [22]. For stratification purposes, we adopted methodologies analogous to those used in social frailty research by Makizako et al [23] and Wei et al [24]. Participants were categorized into 2 cohorts based on their digital isolation index: those scoring 2 or less were classified as “low isolation,” while those scoring 3 or above were designated as “moderate to high isolation.” This bifurcation strategy was designed to elucidate the potential differential impacts of varying degrees of digital isolation on health outcomes.

### Dementia

In the NHATS database, dementia ascertainment is primarily predicated on cognitive function assessments and self-reports or proxy reports. NHATS uses a multifaceted approach to dementia evaluation, encompassing cognitive testing, proxy reports, and clinical records. Specifically, NHATS uses a battery of cognitive tests to assess participants’ memory, attention, and executive function, with these metrics serving as indicators of cognitive status. Concurrently, NHATS collects proxy reports, typically from family members or caregivers, regarding the participants’ cognitive condition. These reports may encompass physician-diagnosed dementia and observe cognitive deficits in activities of daily living. Investigators typically synthesize these data with additional clinical information to determine dementia status and monitor its progression longitudinally. Upon confirmation or report of dementia in any follow-up wave, subsequent inquiries regarding dementia status are discontinued for that participant.

### Covariates

This study incorporated a comprehensive set of covariates to ensure a precise estimation of the association between digital isolation and dementia risk. These covariates encompass sociodemographic characteristics, clinical parameters, and health-related behaviors.

Sociodemographic variables include education level, age, gender, and race or ethnicity. Education level was categorized as <high school, high school or general educational development (GED), some college, and college or above, reflecting participants’ highest educational attainment. Age was stratified into 6 cohorts: 65-69 years, 70-74 years, 75-79 years, 80-84 years, 85-89 years, and ≥90 years. Sex was dichotomized as male or female. Race or ethnicity was categorized as non-Hispanic White, non-Hispanic Black, Hispanic, and other. These variables are routinely used as baseline covariates to adjust for sociodemographic heterogeneity in health outcomes [25-27].

Clinical parameters comprise the number of baseline diseases, depressive symptomatology, and anxiety manifestations. The number of baseline diseases was trichotomized based on self-reported chronic conditions (including arthritis, cardiovascular disease, hypertension, diabetes, pulmonary disease, cerebrovascular accident, osteoporosis, and malignancy): no diseases, 1-2 diseases, and ≥3 diseases. Depressive and anxiety symptoms were assessed by validated self-report instruments and operationalized as binary variables in the analyses [28,29].

Health-related behaviors include smoking status and sleep difficulties. Smoking status was dichotomized as current smokers and noncurrent smokers to account for potential cognitive effects of tobacco use [30]. Sleep difficulties were stratified based on the frequency of difficulty falling asleep within 30 minutes: high sleep difficulty (every night or most nights), moderate sleep difficulty (some nights), and low or no sleep difficulty (rarely or never) [31]. Previous research has demonstrated associations between poor sleep quality, cognitive decline, and elevated dementia risk, warranting its inclusion as a key covariate.

### Statistical Analysis

**Figure 2:**
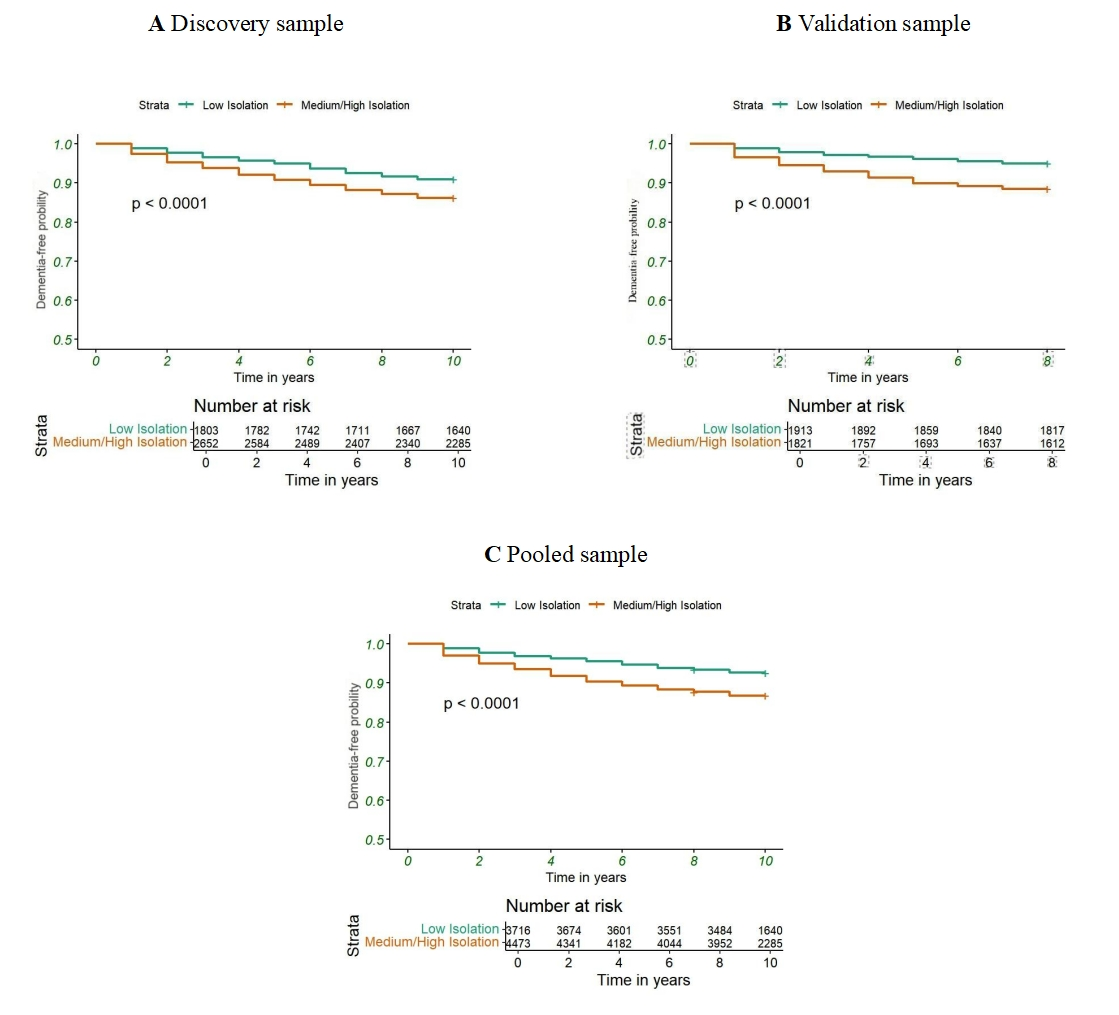
Kaplan‒Meier curves of the relationship between digital isolation and dementia.

To address the complex sampling design of NHATS, we applied person-level sampling weights for longitudinal analyses spanning 2013 to 2022. Clustering and stratification were incorporated by specifying the primary sampling units (PSUs) and strata in the design-based analyses. Baseline characteristics of the study population were summarized using descriptive statistics, with categorical variables reported as frequencies and percentages. Appropriate NHATS survey weights were applied across all analyses to ensure the findings represent the broader US older adult population. To assess the probability of dementia-free survival across different digital isolation groups (low isolation vs moderate to high isolation), we used Kaplan-Meier survival analysis. The resultant Kaplan-Meier survival curves are depicted in Figure 2. To quantify the association between digital isolation and dementia risk, we used Cox proportional hazards regression models, calculating hazard ratios (HRs) with corresponding 95% CIs. A total of 2 models were constructed: model 1 (unadjusted) and model 2 (adjusted for potential confounders including education level, age, gender, race, number of baseline diseases, depression, anxiety, smoking status, and sleep difficulties).

We further investigated the individual components of digital isolation (eg, mobile phone use, computer use, tablet use, frequency of email usage, internet usage, online activities, and health-related online activities) and their respective impacts on dementia risk. Each component was analyzed independently using Cox proportional hazards models to estimate HRs and 95% CIs.

To examine the potential heterogeneity in the association between digital isolation and dementia, we conducted stratified analyses across subgroups defined by gender, age, race or ethnicity, and comorbidity status. Effect modification by these factors was assessed through the inclusion of interaction terms in the Cox models. In addition, a sensitivity analysis was performed to ensure the robustness of our findings. We recalculated the primary models under several alternative conditions, including adjusting the threshold for defining digital isolation, excluding participants with missing key variables, and omitting early dementia cases to reduce potential reverse causality. To enhance the robustness of our findings, we combined the results from the discovery and validation samples using meta-analytic techniques. Pooled HRs and CIs were computed using a fixed-effects model, under the assumption of homogeneity across samples. Statistical analyses were performed using R statistical software (version 4.4.1; R Foundation for Statistical Computing). A 2-sided *P* value <.05 was considered statistically significant for all analyses.

### Ethical Considerations

The study protocol was approved by the institutional review board of Union Hospital, Tongji Medical College, Huazhong University of Science and Technology, Wuhan, China. All methods were conducted in accordance with relevant guidelines and regulations. The NHATS dataset is deidentified and publicly available, with informed consent obtained from participants at enrollment. No additional individual-level consent was necessary for this secondary analysis.

## Results

### Baseline Characteristics of the Study Population

The study cohort comprised 8189 participants, with 4455 individuals in the discovery sample and 3734 in the validation sample. Survey weights provided by NHATS were applied in all analyses to ensure representativeness of the older adult population in the United States. As shown in Table 1, Females constituted a slight majority, accounting for 4724 out of 8189 (57.68%) of the cohort. The racial or ethnic composition was predominantly non-Hispanic White (5615/8189, 68.58%), followed by non-Hispanic Black (1677/8189, 20.48%) and Hispanic (469/8189, 5.73%). Regarding education level, approximately 1250 out of 8189 (15.27%) of participants had less than a high school education, 2560/8189 (31.28%) completed high school or GED, 2375 out of 8189 (29.00%) had some college education, and 2004 out of 8189 (24.45%) had a college degree or higher. Age distribution analysis revealed that the “70-74 years” age group was most prevalent (1906/8189, 23.27%), succeeded by the “75-79 years” (1689/8189, 20.63%) and “80-84 years” (1479/8189, 18.06%) age groups. With respect to baseline diseases, 3918 out of 8189 (47.85%) of participants reported no chronic conditions, while 2593 out of 8189 (31.67%) had 1-2 diseases, and 1678 out of 8189 (20.49%) had 3 or more chronic conditions. The prevalence of depression and anxiety was 2189 out of 8189 (26.74%) and 2819 out of 8189 (34.43%), respectively. The majority of participants (7582/8189, 92.58%) were nonsmokers. Sleep difficulties were reported as significant by 1707 out of 8189 (20.84%) of participants, and moderate by 1989 out of 8189 (24.29%). In the context of digital isolation, 4473 out of 8189 (54.62%) of participants were categorized as experiencing moderate to high isolation, while 3716 out of 8189 (45.38%) were classified as having low isolation. Digital device use rates were as follows: mobile phones (6615/8189, 80.8%), computers (4857/8189, 59.31%), and tablets (2045/8189, 24.98%). Frequent engagement in digital activities was reported for email or text messaging 6537/8189, (79.83%), internet access (4709/8189, 57.51%), general online activities (7282/8189, 88.93%), and health-related online activities (6315/8189, 77.13%).

**Table 1:** Baseline characteristics of the study population.

Variables		Discovery sample (N=4455)	Validation sample (N=3734)	Pooled sample (N=8189)
**Gender, n (%)**				
	Male	1843 (41.37)	1622 (43.44)	3465 (42.32)
	Female	2612 (58.63)	2112 (56.56)	4724 (57.68)
**Race or ethnicity^a^, n (%)**				
	White, non-Hispanic	3162 (70.98)	2453 (65.69)	5615 (68.58)
	Black, non-hispanic	915 (20.54)	762 (20.41)	1677 (20.48)
	Hispanic	230 (5.16)	239 (6.4)	469 (5.73)
	Other	148 (3.32)	280 (7.5)	428 (5.22)
**Education level, n (%):**				
	< High school	680 (15.26)	570 (15.27)	1250 (15.27)
	High school or GED	1380 (31)	1180 (31.6)	2560 (31.28)
	Some college	1370 (30.77)	1005 (26.91)	2375 (29)
	College or above	1025 (23)	979 (26.22)	2004 (24.45)
**Age (years), n (%)**				
	65 to 69	459 (10.3)	1000 (26.78)	1459 (17.82)
	70 to 74	1076 (24.15)	830 (22.23)	1906 (23.27)
	75 to 79	961 (21.57)	728 (19.5)	1689 (20.63)
	80 to 84	899 (20.18)	580 (15.53)	1479 (18.06)
	85 to 89	647 (14.52)	355 (9.51)	1002 (12.23)
	90 or above	413 (9.27)	241 (6.45)	654 (7.99)
**Baseline disease, n (%)**				
	No disease	3568 (80.09)	350 (9.37)	3918 (47.85)
	1-2 diseases	861 (19.33)	1732 (46.38)	2593 (31.67)
	3 or more diseases	26 (0.58)	1652 (44.24)	1678 (20.49)
**Depression^b^, n (%)**				
	No depression	3241 (72.75)	2759 (73.89)	6000 (73.26)
	Depression	1214 (27.25)	975 (26.11)	2189 (26.74)
**Anxiety, n (%)**				
	No anxiety	2908 (65.27)	2462 (65.93)	5370 (65.57)
	Anxiety	1547 (34.73)	1272 (34.07)	2819 (34.43)
**Smoking status, n (%)**				
	Nonsmoker	4152 (93.2)	3430 (91.86)	7582 (92.58)
	Smoker	303 (6.8)	304 (8.14)	607 (7.42)
**Sleep difficulty^c^, n (%)**				
	High difficulty	848 (19.13)	859 (23.07)	1707 (20.84)
	Medium difficulty	1115 (25.15)	874 (23.47)	1989 (24.29)
	Low or no difficulty	2470 (55.72)	1991 (53.46)	4461 (54.47)
**Digital isolation^d^ group, n (%)**				
	Low isolation	1803 (40.47)	1913 (51.23)	3716 (45.38)
	Medium and high isolation	2652 (59.53)	1821 (48.77)	4473 (54.62)
**Items of digital isolation, n (%)**				
	Phone use, n (%)	3446 (77.35)	3169 (84.87)	6615 (80.8)
	Computer use, n (%)	2531 (56.81)	2326 (62.29)	4857 (59.31)
	Tablet use, n (%)	764 (17.15)	1281 (34.31)	2045 (24.98)
	Email frequency, n (%)	3603 (80.88)	2934 (78.58)	6537 (79.83)
	Internet use, n (%)	2498 (56.07)	2211 (59.21)	4709 (57.51)
	Online activity, n (%)	3945 (88.55)	3337 (89.37)	7282 (88.93)
	Health-related online use, n (%)	3484 (78.2)	2831 (75.82)	6315 (77.13)

^a^Race or ethnicity was reclassified into 4 categories: White, non-Hispanic; Black, non-Hispanic; Hispanic; and Other.

^b^The depression, anxiety, and smoking status variables were reclassified as binary variables. Responses of “Don't know,” “Refused to answer,” and similar were treated as missing data.

^c^Sleep difficulty was categorized by the frequency of difficulty falling asleep within 30 minutes, with unknown or refused responses treated as missing data.

^d^The Digital Isolation Index was used to create a new variable (Digital Isolation Group) dividing participants into low and moderate and high isolation groups.

### Association Between Digital Isolation and the Risk of Dementia

The association between digital isolation and dementia risk was comprehensively examined, adjusting for factors including education level, age, gender, race or ethnicity, number of baseline diseases, depression, anxiety, smoking status, and sleep difficulties, with results presented in Table 2. In the discovery sample, the moderate to high isolation group demonstrated a significantly elevated risk of dementia compared with the low isolation group. The unadjusted Cox proportional hazards model (model 1) yielded a HR of 1.58 (95% CI 1.31-1.89, *P*<.001) for dementia in the moderate to high isolation group, indicating a 58% higher relative risk of dementia compared with the low isolation group. After adjusting for potential confounders (age, gender, race or ethnicity, number of baseline diseases, depression, anxiety, smoking status, and sleep disorders), the HR in model 2 attenuated to 1.22 (95% CI 1.01-1.47, *P*=.041), yet remained statistically significant. The validation sample corroborated these findings. The unadjusted model 1 revealed an HR of 2.36 (95% CI 1.86-3.01, *P*<.001) for the moderate to high isolation group, while the adjusted model 2 showed an HR of 1.62 (95% CI 1.27-2.08, *P*<.001), consistently indicating a significantly higher dementia risk in the moderate to high isolation group.

**Table 2:** Association between digital isolation and the risk of dementia.

Sample and variables			Event, n/N (%)		Model 1^a^ HR^b^ (95% CI)		*P* value		Model 2^c^ HR (95% CI)		*P* value
**Discovery sample**											
	Low isolation (reference range)	1803/4455 (40.47)		1 (Ref)		—^d^		1 (Ref)		—	
	Medium and high isolation	2652/4455 (59.53)		1.58 (1.31-1.89)		<.001		1.22 (1.01-1.47)		.04	
**Validation sample**											
	Low isolation (reference range)	1913/3734 (51.23)		1 (Ref)		—		1 (Ref)		—	
	Medium and high isolation	1821/3734 (48.77)		2.36 (1.86-3.01)		<.001		1.62 (1.27-2.08)		<.001	
**Pooled sample**											
	Low isolation (reference range)	3716/8189 (45.38)		1 (Ref)		—		1 (Ref)		—	
	Medium and high isolation	4473/8189 (54.62)		1.89 (1.63-2.19)		<.001		1.36 (1.16-1.59)		<.001	

^a^Model 1: unadjusted Cox proportional hazards model.

^b^HR: hazard ratio.

^c^Model 2: Cox proportional hazards model adjusted for age, gender, race, education level, baseline disease, depression, anxiety, smoking status, and sleep difficulty.

^d^Not applicable.

To enhance the robustness of our findings, we applied meta-analytic techniques to pool results from both samples. The pooled analysis yielded HRs of 1.89 (95% CI 1.63-2.19, *P*<.001) in the unadjusted model 1 and 1.36 (95% CI 1.16-1.59, *P*<.001) in the adjusted model 2, further substantiating the significant risk increase.

**Figure 3:**
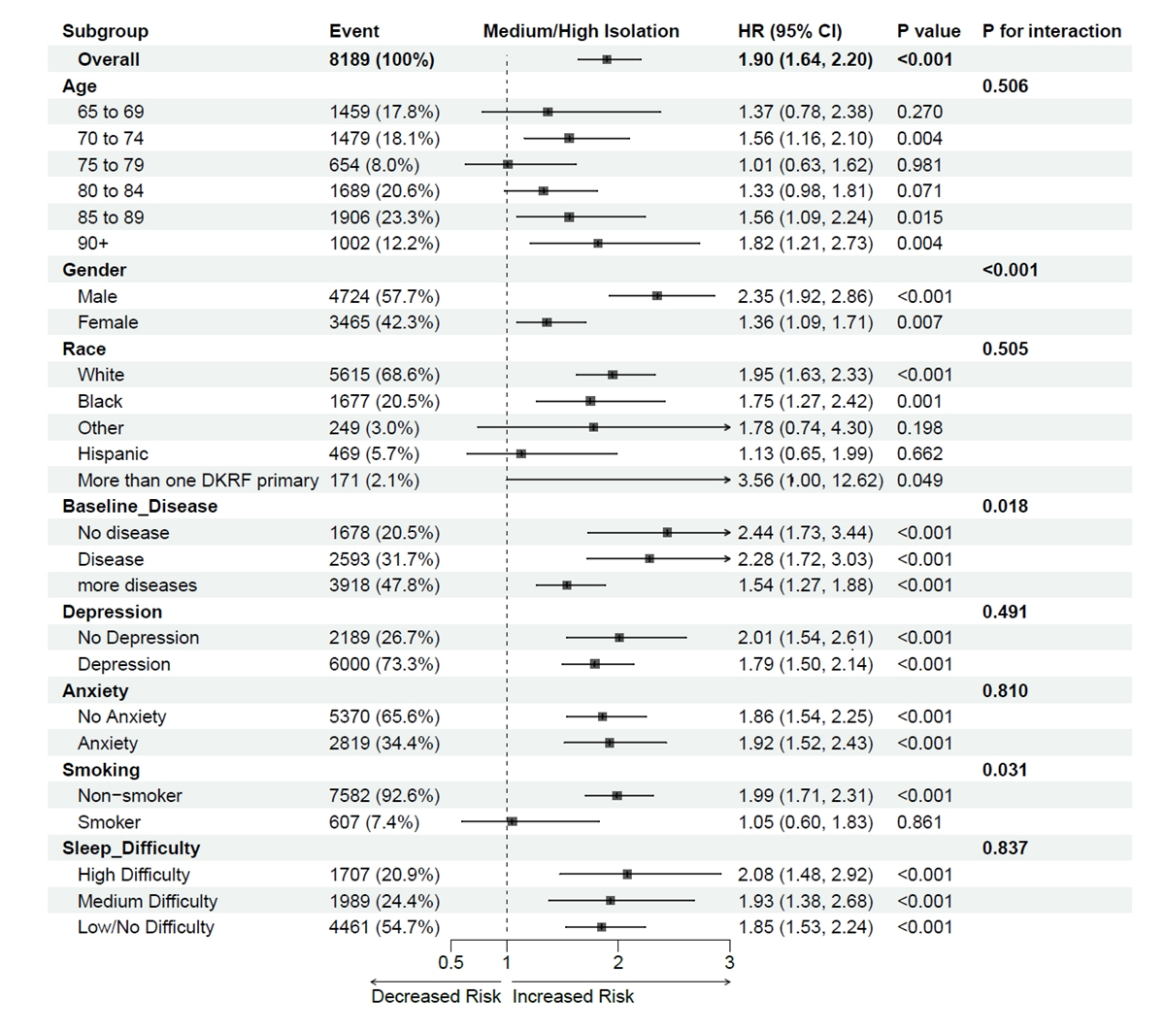
Association between digital isolation and risk of dementia among subgroups. Reference group: Low digital isolation group. All models were adjusted for age, gender, race, baseline disease, depression, anxiety, smoking, sleep difficulty. P-int represents the heterogeneity among subgroups based on the metaregression analysis.

Kaplan-Meier survival curves were used to visualize dementia incidence across different digital isolation groups (Figure 2). These curves demonstrated a significantly higher probability of dementia development in the moderate to high isolation group, corroborating the Cox regression analysis results. Figure 3 illustrates the association between digital isolation and dementia risk across various demographic and clinical subgroups. Consistently elevated dementia risk was observed in the moderate to high isolation group across all subgroups, with notable differences in gender, age, and comorbidity status. These findings underscore the pervasive impact of digital isolation on dementia onset, demonstrating a consistent trend of increased risk both in the overall population and within specific subgroups.

### Association Between Digital Isolation Components and the Risk of Dementia

We investigated the association between individual components of digital isolation and incident dementia risk, with results summarized in Table 3. In the discovery sample, we analyzed the use of mobile phones, computers, tablets, and the internet in relation to dementia risk. The unadjusted Cox proportional hazards model (model 1) revealed that nonusers of mobile phones had a significantly higher dementia risk compared with users (HR 1.78, 95% CI 1.41-2.24, *P*<.001). After adjusting for covariates (model 2), the HR attenuated to 1.45 (95% CI 1.14-1.84, *P*=.002). For computer use, the adjusted HR was 1.15 (95% CI 0.96-1.38, *P*=.12), approaching but not reaching statistical significance. Similarly, adjusted HRs for tablet use were not statistically significant across samples, with a pooled HR of 1.05 (95% CI 0.78-1.42, *P*=.73).

**Table 3:** Association between digital isolation components and the risk of dementia.

Component and variables	Discovery sample				Validation sample				Pooled sample			
	Model 1^a^, HR^b^ (95% CI)	*P* values	Model 2^c^, HR (95% CI)	*P* values	Model 1, HR (95% CI)	*P* values	Model 2, HR (95% CI)	*P* values	Model 1, HR (95% CI)	*P* values	Model 2, HR (95% CI)	*P* values
Phone use (nonuse vs use)	1.78 (1.41-2.24)	<.001	1.45 (1.14-1.84)	.002	3.05 (2.28-4.08)	<.001	2.12 (1.55-2.9)	<.001	2.05 (1.6-2.62)	<.001	1.65 (1.35-2.02)	<.001
Computer use (nonuse vs use)	1.22 (1-1.5)	.052	1.10 (0.88-1.36)	.386	1.50 (1.15-1.97)	.003	1.28 (0.98-1.66)	.071	1.25 (0.98-1.59)	.074	1.15 (0.96-1.38)	.118
Tablet use (nonuse vs use)	1.39 (1.02-1.88)	.035	1.28 (0.94-1.73)	.108	1.19 (0.88-1.6)	.264	1.10 (0.82-1.48)	.503	1.10 (0.8-1.53)	.548	1.05 (0.78-1.42)	.734
Email frequency (nonuse vs use)	0.74 (0.5-0.99)	.043	0.78 (0.58-1.04)	.090	1.03 (0.74-1.44)	.848	1 (0.72-1.38)	.998	0.95 (0.7-1.28)	.707	0.9 (0.66-1.23)	.51
Internet use (nonuse vs use)	1.42 (1.12-1.79)	.004	1.28 (1-1.64)	.047	1.75 (1.3-2.34)	<.001	1.56 (1.15-2.11)	.005	1.6 (1.24-2.07)	<.001	1.42 (1.15-1.76)	.001
Online activity (nonuse vs use)	1.12 (0.78-1.59)	.541	1.1 (0.77-1.57)	.612	1.93 (1.24-3.02)	.004	1.78 (1.14-2.79)	.012	1.6 (1.1-2.31)	.013	1.32 (1-1.74)	.048
Health-related online use (nonuse vs use)	1.08 (0.78-1.48)	.656	1.15 (0.83-1.59)	.402	0.92 (0.61-1.4)	.707	0.9 (0.58-1.36)	.594	0.95 (0.7-1.28)	.745	0.9 (0.64-1.28)	.576

^a^Model 1: unadjusted Cox proportional hazards model.

^b^HR: hazard ratio.

^c^Model 2: Cox proportional hazards model adjusted for age, gender, race, education level, baseline disease, depression, anxiety, smoking status, and sleep difficulty.

In the validation sample, nonusers of mobile phones exhibited a significantly higher dementia risk, with an unadjusted HR of 3.05 (95% CI 2.28-4.08, *P*<.001), which decreased to 2.12 (95% CI 1.55-2.9, *P*<.001) after adjustment. Nonusers of the internet demonstrated a robust association with dementia risk, with a pooled adjusted HR of 1.42 (95% CI 1.15-1.76, *P*=.001). For individuals not engaging in online activities, the pooled adjusted HR was 1.32 (95% CI 1-1.74, *P*=.05), further underscoring the importance of digital engagement. However, nonusers of health-related online platforms showed no significant association with dementia risk (pooled HR 0.9, 95% CI: 0.64-1.28, *P*=.58).

These findings underscore that specific digital behaviors, particularly mobile phone and internet use, are significantly associated with a lower risk of dementia. In contrast, the influence of other digital components, such as computer or tablet use, appears less consistent or pronounced.

### Sensitivity Analyses

To evaluate the robustness of our findings and address potential confounders, we conducted several sensitivity analyses. First, we repeated the Cox regression models after excluding participants with incomplete education data, confirming that the significant association between digital isolation and dementia risk remained robust even after adjusting for educational level (adjusted HR range 1.20-1.45, all *P*<.05). Second, we applied alternative thresholds for defining digital isolation, such as categorizing digital isolation index ≥4 as high isolation, and observed results consistent with the primary analysis. Third, we excluded individuals who developed dementia or died within the first 2 years of follow-up to reduce potential reverse causation, finding HRs comparable with those in the main analysis (HR changes<10%). Finally, we replicated the main models without applying survey weights. Although the unweighted models yielded slightly smaller standard errors, the overall trends remained consistent. Collectively, these sensitivity analyses indicate that our key conclusions are robust across varying analytical assumptions and sample selections.

## Discussion

### Principal Results

This study examined the association between digital isolation and dementia risk, demonstrating that higher levels of digital isolation significantly increase the risk of dementia. These findings emphasize the critical role of digital engagement in promoting cognitive health among older adults and provide valuable insights for public health policy development. By using the nationally representative NHATS dataset and its survey weights, we ensured that our results reflect the broader US older adult population, thereby enhancing the robustness of our conclusions. Our analysis identified a significant relationship between digital isolation and dementia risk. In both discovery and validation samples, individuals who did not use basic digital devices (eg, mobile phones and the internet) or who lacked online activity participation exhibited a substantially higher dementia risk compared with their digitally engaged peers. Specifically, nonusers of mobile phones had a 1.65-fold increased risk (95% CI 1.35-2.02, *P*<.001), while nonusers of the internet demonstrated a 1.42-fold increased risk (95% CI 1.15-1.76, *P*=.001) in the pooled analysis. In addition, the pooled analysis showed that individuals with a higher digital isolation index faced a 1.36-fold increased risk of dementia (95% CI 1.16-1.59, *P*<.001). These results are consistent with previous research identifying social isolation and lack of social interaction as independent risk factors for dementia [13,32].

However, some digital activities, such as emailing or texting, showed no significant association with dementia risk. This might be because these activities are less interactive or infrequently used by older adults, offering limited cognitive or social stimulation compared with more engaging digital behaviors, such as frequent phone or internet use.

Furthermore, this study validates digital isolation as a novel form of isolation that may influence cognitive health through multiple mechanisms. Digital technologies can promote social interaction and participation, crucial for maintaining cognitive function [33-35]. Conversely, a lack of digital engagement may exacerbate social isolation, thereby increasing cognitive decline risk. In addition, digital technology use can provide cognitive stimulation, potentially helping older adults maintain brain vitality [36,37]. Thus, digital isolation not only reflects diminished social interaction but may also represent insufficient cognitive stimulation. These findings have significant public health implications. As supported by recent studies, educational attainment can significantly influence individuals’ ability to engage with digital tools, highlighting the importance of prioritizing older adults with lower educational backgrounds in targeted interventions [38-40]. As society undergoes digital transformation, issues related to digital engagement among older adults are becoming increasingly salient. Our study suggests that promoting digital technology use among older adults, particularly within vulnerable populations, could effectively mitigate dementia risk. This implies that enhancing digital literacy and expanding access to digital resources should be integral components of dementia prevention strategies. While other studies may not have used the specific “digital isolation index,” research examining older adults’ online engagement or technology use consistently demonstrates that digitally engaged older adults report better cognitive and psychosocial outcomes [10,41,42]. These findings reinforce the protective role of digital engagement in cognitive health and underscore the importance of encouraging digital technology adoption among older adults.

### Limitations

However, this study has limitations. Despite controlling for multiple covariates, the observational nature of our study design precludes complete elimination of confounding factors. For instance, individuals’ health behaviors and cognitive abilities may simultaneously influence their digital technology use and dementia risk, potentially leading to reverse causality [19,43]. Furthermore, educational attainment could discourage older adults from adopting digital devices, complicating the interpretation of whether digital isolation is a cause or result of declining cognitive function. In addition, our reliance on self-reported data may introduce information bias, particularly in the preclinical stages of dementia when individuals’ recollection of their digital usage may not be entirely accurate.

Notwithstanding these limitations, this study provides important empirical support for exploring the relationship between digital isolation and dementia risk. Future research should further investigate factors that promote or hinder digital engagement among older adults to develop more effective interventions. In addition, prospective longitudinal studies, randomized controlled trials, and qualitative investigations are crucial for further validating our findings and examining the causal relationship between digital technology use and cognitive health.

### Conclusions

This study, examining the association between digital isolation and dementia risk in older adults, underscores the critical role of digital engagement in maintaining cognitive health. Our findings demonstrate that digital isolation significantly increases dementia risk, particularly among individuals who do not use basic digital devices, lack online communication, and abstain from online activities. These findings indicate that strategies focused on enhancing digital literacy, promoting equitable access to digital tools, and addressing educational disparities may effectively mitigate dementia risk in an increasingly digitalized society.

Our research not only provides new evidence of the detrimental effects of digital isolation but also offers valuable insights for future public health interventions. For instance, community-based digital literacy programs and targeted technology access initiatives could help reduce digital isolation among older adults, especially those from underserved or low-education backgrounds. Enhancing digital literacy and expanding accessibility to digital resources among older adults may effectively reduce digital isolation, potentially lowering dementia incidence. Future research should explore the specific mechanisms through which digital engagement affects cognitive health and further validate the causal relationship between digital isolation and dementia through longitudinal studies and intervention trials.

In conclusion, this study highlights the importance of digital technology in older adults’ health and advocates for the integration of digital literacy education, user-friendly technological interfaces, and widespread digital resource availability in public health policies. These measures will facilitate older adults’ integration into the digital society, thereby potentially improving their cognitive health and overall quality of life. Ensuring that these interventions are adapted to individual educational levels may be pivotal for maximizing their preventive impact.

## Abbreviations

GED: general educational development
HR: hazard ratio
NHATS: National Health and Aging Trends Study
PSU: primary sampling unit
